# Treatment of Plethora by Saline Medicines

**Published:** 1845-02

**Authors:** 


*Treatment of Plethora by Saline Medicines.*—"The treatment of plethora is often not nearly so easy as that of anæmia. In many cases it will not suffice merely to abstain from animal food, and to drink large quantities of simple cooling beverages, in the hope of attenuating and impoverishing the condition of the blood. Then, again, the effects of bloodletting are generally only transitory; aud, moreover, the very loss of blood seems not unfrequently to induce a more active proportionate formation of it. On the whole, the use of saline laxatives, and of the hydrochlorate of ammonia (sal ammoniac), seem to be the most useful means that can be employed for the relief of plethora, when it gives rise to inconvenient symptoms.

"Dr. Lheritier, in his recent treatise on pathological chemistry, informs us that he has found that the proportion of the red globules in the blood of rabbits was decidedly modified by the internal use of this salt, in the course of two or three weeks.

"The nitrate of potash has similar effects; so also have the alkaline subcarbonates, and the liquor potassæ itself. Perhaps the latter is, on the whole, the most efficient impoverisher of the blood, provided, also, the diet is spare, and not too nutritious, and all malt liquors are avoided."—*Medico-Chirurgical Review.*

